# Modifications of Gut Microbiota after Grape Pomace Supplementation in Subjects at Cardiometabolic Risk: A Randomized Cross-Over Controlled Clinical Trial

**DOI:** 10.3390/foods9091279

**Published:** 2020-09-11

**Authors:** Sara Ramos-Romero, Daniel Martínez-Maqueda, Mercè Hereu, Susana Amézqueta, Josep Lluís Torres, Jara Pérez-Jiménez

**Affiliations:** 1 Institute of Advanced Chemistry of Catalonia (IQAC-CSIC), Jordi Girona 18-26, 08034 Barcelona, Spain; sara.ramosromero@ub.edu (S.R.-R.); merce.hereu@iqac.csic.es (M.H.); joseplluis.torres@iqac.csic.es (J.L.T.); 2 Department of Cell Biology, Physiology & Immunology, Faculty of Biology, University of Barcelona, Avinguda Diagonal 643, 08028 Barcelona, Spain; 3 Department of Metabolism and Nutrition, Institute of Food Science, Technology and Nutrition (ICTAN-CSIC), José Antonio Novais 10, 28040 Madrid, Spain; daniel.martinez.maqueda@madrid.org; 4 Departament d’Enginyeria Química i Química Analítica, Institut de Biomedicina (IBUB), Universitat de Barcelona, Carrer de Martí i Franquès, 1-11, 08028 Barcelona, Spain; samezqueta@ub.edu

**Keywords:** metabolic syndrome, microbiota, insulin sensitivity, polyphenols, grape pomace

## Abstract

Polyphenols are dietary bioactive compounds able to induce modifications in the gut microbiota profile, although more clinical studies are needed. With this aim, a randomized cross-over clinical trial was conducted, where 49 subjects at cardiometabolic risk (exhibiting at least two metabolic syndrome factors) were supplemented with a daily dose of 8 g of grape pomace (GP) for 6 weeks, with an equivalent control (CTL) period. The levels of total bacteria and Bacteroidetes, Firmicutes, Lactobacilliales, *Bacteroides* and *Prevotella* were estimated in fecal DNA by quantitative real-time PCR (qPCR), while fecal short-chain fatty acids (SCFAs) were assessed by gas chromatography. Several cardiometabolic markers were evaluated in blood samples. GP reduced insulin levels only in half of the participants (responders). GP supplementation did not cause significant modifications in the microbiota profile of the whole group, except for a tendency (*p* = 0.059) towards a decrease in the proportion of Lactobacilliales, while it increased the proportion of *Bacteroides* in non-responder subjects. The reduction of insulin levels in subjects at cardiometabolic risk upon GP supplementation appears not to be induced by changes in the major subgroups of gut microbiota. Further studies at the species level may help to elucidate the possible role of microbiota in GP-induced insulinemic status.

## 1. Introduction

Colonic microbiota has emerged as a key player in the crosstalk between diet and health. Increasing evidence connects unbalances in gut microbiota (dysbiosis) with pathologies, such as cancer [1] or Parkinson’s disease [2]. Dysbiosis appears to be particularly decisive in the development of risk factors for cardiometabolic disease. It has been shown that the profile of gut microbiota in subjects presenting obesity [3], impaired glucose tolerance [4], or hypertension [5] are different to those of healthy subjects. Metabolic syndrome (MetS) is a combination of cardiometabolic risk factors (central obesity, high blood glucose and triglycerides, low HDL cholesterol, hypertension) arising from underlying pathophysiological processes such as insulin resistance (IR) [6], sub-clinical inflammation [7], or oxidative stress (OS) [8]. Since the prevalence of MetS is increasing, there is growing interest in further exploring the role of dysbiosis as well as the opportunities gut microbiota may offer for dietary interventions [9].

Polyphenols, a wide group of secondary metabolites widely distributed in foods of vegetal origin, may be effective against MetS-associated risk factors, particularly low HDL-cholesterol [10]. More robust clinical trials are still needed [11]. Polyphenols may exert their action by different mechanisms: improvement of insulin and adiponectin signaling pathways; inhibition of inflammation signaling pathways; stimulation of endogenous antioxidant systems; repression of intestinal lipid absorption; decrease of triglyceride content in skeletal muscle; inhibition of chylomicron/VLDL secretion; stimulation of nitric oxide production [12,13,14,15,16,17,18,19]. Polyphenols may exert some of their effects through the modulation of oral and gut microbiota. The limited evidence currently available from in vitro, pre-clinical and clinical studies is suggesting that some of the effects of polyphenols against colorectal cancer and cardiovascular disease may be related to changes in the relative populations of bacterial subgroups such as *Bacteriodes*, *Lactobacillus*, and *Bifidobacterium* [20,21].

Another important aspect in the health effects of dietary bioactive compounds is the inter-individual variability. Not all subjects exhibit the same response to supplementation (e.g., with polyphenols) [22]. The action of microbiota is probably a major determinant of inter-individual variability as polyphenols are extensively transformed by several bacterial species, which generate a great variety of bioactive absorbable metabolites [23]. Indeed, the concept of metabotypes, i.e., groups of subjects with a tendency to originate certain combinations of circulating polyphenol metabolites as compared to other groups not able to generate them, has become a key concept in the field of dietary polyphenols [24].

Grape pomace (GP) is generated during wine making being commonly discarded or underutilized. This product is very rich in both dietary fiber and polyphenols (particularly the so-called non-extractable polyphenols, associated with dietary fiber [25]), thus, being suggested as a new food ingredient in multiple products [26]. Indeed, some studies have reported that the intake of GP or derived extracts improved blood pressure, fasting glucose, fasting or postprandial insulin and lipid profile [27,28,29,30,31]. Several in vitro, preclinical, and clinical studies have evaluated the changes induced by different grape-derived products in the populations of gut microbiota as well as their associated beneficial effects [32]. A clinical trial with healthy women supplemented with a GP extract for 3 weeks did not find any modification in gut microbiota profile [28]. As far as we know, no clinical trial has explored whether the sustained intake of whole GP may affect microbiota profile.

Dried GP supplementation at nutritional doses (8 g daily) for 6 weeks in subjects at high cardiometabolic risk led to a significant improvement in insulin sensitivity in a randomized cross-over clinical trial [29]. This study explored the potential modifications in microbiota profile induced by GP supplementation, exploring specifically the possible ways in which GP supplementation might have differentially induced a reduction of insulin secretion in responder individuals, with particular attention to changes in representative gut bacterial populations.

## 2. Experimental Materials

### 2.1. Composition of Grape Pomace

In this study, subjects were daily supplemented with freeze-dried and milled GP (*Vitis vinifera* L. cv. Tempranillo) provided in monodoses (8 g). To ensure safety, standard microbiological and metal analysis were performed in the product.

Detailed composition of the product has been provided elsewhere [29]. Its main constituent was dietary fiber (68.2%), being insoluble fraction a 98% of it. The next major constituent were polyphenols (29.6%), with 66% of them being non-extractable polymeric proanthocyanidins. Protein content was 11.6%, fat content was 10.3% and ash content was 5.7%. Simple sugars content in GP was 2.5%.

This study was a randomized cross-over controlled clinical trial. Permissions were obtained from the Ethics Subcommittee of the Spanish National Research Council (CSIC), Madrid, Spain (2016/12/13), and the Ethics Committee for Clinical Research of the University Hospital Puerta de Hierro-Majadahonda, Majadahonda, Spain (2016/12/02). It was registered in the Clinical Trials database with the identifier NCT03076463, where whole trial protocol is available. Recruitment for the study took place between December 2016 and March 2017, while the study was conducted between March 2017 and July 2017. A written informed consent was requested to participate in the study.

Details on this clinical trial have been already reported, together with CONSORT flow diagram [29]. Briefly, participants were aged 18–70 years with at least two of the factors requested for MetS diagnose [33], without diagnosis or medication for cardiometabolic pathologies. Exclusion criteria were: pregnancy, lactation, current or close participation in any other dietary intervention study. Although it was not an exclusion criterion, subjects were asked about dietary supplement consumption and none of them was consuming probiotics. The number of subjects was fixed in 50; for this, a 30% decrease in HOMA-IR (homeostatic model assessment for insulin resistance) index was fixed as primary outcome, based on previous nutritional clinical trials [34]. For a statistical power of 95% and an alpha value of 0.05, a number of 40 subjects was obtained, which was increased to 50 foreseeing potential withdrawals from the study. Subject were recruited by mailing and public advertisement in the Madrid region.

The study had a cross-over design, where subjects were randomly assigned (based on recruitment date) either to supplementation with GP (suspended in water) or to the C arm (since no valid placebo was found for this period, they followed their usual diet and had the same controls as in the GP period). Each period had a duration of 6 weeks, separated by a 4 week wash-out. At the beginning of the GP period, the subjects received the doses for the six weeks and were instructed to keep them frozen, as well as not to modify their dietary habits. Nevertheless, three days before each visit (at the beginning and at the end of each period) they had to follow a low polyphenol diet, for which specific instructions were provided. Random allocation sequence, participant recruitment and participant assignment to interventions were performed by the same researchers.

When each period started and finished, fasting blood and urine samples were collected. In these visits, the volunteers also provided a fecal sample from the previous 24 h. Additionally, at the beginning and at the end of the GP period, half of the participants were subjected to a fasting OGTT (oral glucose tolerance test). After ingesting 75 g of glucose in 200 mL of water, blood samples were collected at 0, 30, 60, and 120 min. Centrifugation was applied at 1000 *g* for 15 min in order to obtain serum and plasma samples. All biological samples were stored at −80 °C. Fasting samples were used to assess the primary outcome, as well as the other cardiometabolic markers described below. Postprandial samples were used to evaluate glucose and insulin concentration.

Once the study was finished, and after observing that GP caused a significant decrease in fasting plasma insulin, a more detailed analysis of data for this parameter was performed. It was found that in 23 of the subjects this reduction was of at least 10% (responders, GP-R) while in 26 of the subjects there was an increase (non-responder, GP-NR). This classification was then used to evaluate the modifications in other cardiometabolic markers, microbiota composition and fecal short-chain fatty acids (SCFAs).

### 2.2. Cardiometabolic Markers

The markers measured by an automatic analyzer (ADVIA Chemistry XPT System, Siemens Healthineers, Tarrytown, NY, USA) were: serum total cholesterol, HDL cholesterol, LDL cholesterol, triglycerides and high-sensitive plasma C reactive protein. Plasma insulin was determined by a commercial ELISA kit (Merck-Millipore, Burlington, MS, USA), according to the manufacturer instructions. Commercial kits were used for plasma and urine uric acid (Spinreact S.A., Sant Esteve de Bas, Spain) and urine creatinine concentration (Cromatest Linear Chemicals S.L., Montgat, Spain), used to normalize urinary determinations. The enzyme electrode method was used to assess blood glucose (in fasting and postprandial state) with a Free Style Optimum Neo blood glucose meter from Abbott (Chicago, IL, USA).

For blood pressure, mean values for two measurements made between 8:00 and 10:00 h in a quiet temperature-controlled room using an automated digital oscillometric device (Omron model M6 Comfort, Omron Corporation, Tokyo, Japan) were obtained. Height, body weight, and abdominal perimeter were measured. Total body fat and abdominal fat were assessed by using a tetrapolar bioimpedance system (Tanita BC601, Arlington heights, IL, USA).

### 2.3. Fecal Microbiota

The levels of total bacteria and Bacteroidetes, Firmicutes, Lactobacilliales, and *Bacteroides* and *Prevotella* were estimated from fecal DNA by quantitative real-time PCR (qPCR). First, a DNA extraction from the feces was performed using QIAamp DNA StoolMini Kit from Qiagen (Hilden, Germany) and then a Nanodrop 8000 Spectrophotometer (Thermo Scientific, Waltham, MA, USA) was used for quantification. A dilution to 20 ng/µL was performed in all DNA samples. A LightCycler 480 II (Roche, Basel, Switzerland) was employed for the qPCR experiments, performed in triplicate. The samples contained DNA (2 µL) and a master mix (18 µL) made of 2XSYBR (10 µL), the corresponding forward and reverse primer (1 µL each), and water (6 µL). All reactions were paralleled by analysis of a non-template control (water) and a positive control. The identified regions of the 16S rRNA gene were previously tested [35,36,37,38,39]. They were predicted to be highly conserved amongst each studied bacterial subgroup and specific for them. The primers and annealing temperatures are detailed in Table 1.

The qPCR cycling conditions were: 10 s at 95 °C, then 45 cycles of 5 s at 95 °C, 30 s at primer-specific annealing temperature (Table 1), and 30 s at 72 °C (extension). Next to amplification, the specificity of the qPCR was evaluated, for which melting curve analysis was carried out by treatment for 2 s at 95 °C, 15 s at 65 °C followed by continuous increase of temperature up to 95 °C (0.11 °C/s) with five fluorescence recordings per degree Celsius. The relative DNA abundances for the different genes were calculated from the second derivative maximum of their respective amplification curves (Cp, calculated in triplicate) by considering Cp values to be proportional to the dual logarithm of the inverse of the specific DNA concentration. For this, this equation: was applied: [DNAa]/[DNAb] = 2^Cpb-Cpa^ [40]. Total bacteria was normalized as 16S rRNA gene copies per mg of wet feces (copies per mg).

### 2.4. Fecal Short-Chain Fatty Acids

SCFAs were analyzed in feces by gas chromatography. For this, a previously described method [39] was applied, incorporating several validated changes. First, the freeze-dried fecal samples were weighed (~50 mg dry matter) and a solution (1.5 mL) providing the internal standard 2-ethylbutiric acid (6.67 mg/L) and oxalic acid (2.97 g/L) in acetonitrile/water 3:7 was added. Then, SCFAs were extracted for 10 min using a rotating mixer. The suspension was centrifuged (5 min, 12,880 g) in a 5810R centrifuge (Eppendorf, Hamburg, Germany) and the supernatant filtered through a 0.45 µm nylon filter. From this, an aliquot (0.7 mL) was diluted with acetonitrile/water 3:7 up to 1 mL. The equipment used for SCFAs analysis was a Trace 2000 gas chromatograph coupled to a flame ionization detector (ThermoFinnigan, Waltham, MA, USA). The column was an Innowax 30 m × 530 µm × 1 µm capillary column (Agilent, Santa Clara, CA, USA). Chrom-Card software was used for data analysis. 

### 2.5. Statistical Analysis

Graph Pad Prism 5 (Graph Pad Software, Inc., San Diego, CA, USA) software was used for statistical analysis. Results are provided as mean values accompanied by standard errors (SEM). For cardiometabolic markers, the variations in NR and R subjects were compared by Kruskal-Wallis test, since they did not follow a normal distribution. For microbiota composition and fecal SCFAs, normal distributions and heterogeneity of the data were evaluated, and the statistical significance was determined by Student’s *t* test. Differences were considered significant when *p* < 0.05.

## 3. Results

### 3.1. Subject Characteristics

The study was completed by a total of 49 participants aged 20–65 with a mean age of 42.6 (SEM 1.6). The proportion of male participants was 55%. Since they must fulfill a minimum of two MetS factors, it was found that 63% already fulfilled all criteria for official MetS diagnoses. Besides overweight/obesity, 67% of the subjects exhibited hypertension and 57% of them presented glycaemia (both factors were present in 24% of total population). The less common MetS factor was hypertriglyceridemia, found in 12% of the subjects. Basal individual values for MetS factors are provided as Supplementary Material Table S1.

### 3.2. Evolution in Cardiometabolic Markers

No significant differences in the cardiometabolic markers were observed during the control (CTL) period (data not shown). Regarding the GP period, basal values for the different cardiometabolic markers, together with the variation observed in NR and R subjects, are shown in Table 2. As expected, significant differences (*p* < 0.00001) were observed between NR and R subjects for plasma insulin values, since this was the criterium (insulin decrease of at least 10% due to GP supplementation) used to classify the subjects as R or NR. In R subjects, median variation (−42%) was close to mean value, while in NR subjects, the median variation (49.3%) was far from mean value, evidencing a higher dispersion in the data. Nevertheless, the mean and median increase in NR subjects would show that, in this group, insulin was not only not reduced by GP supplementation, but even increased by it. For the other parameters, significant differences were not found between R and NR subjects.

### 3.3. Fecal Microbiota and SCFAs

**Figure 1:**
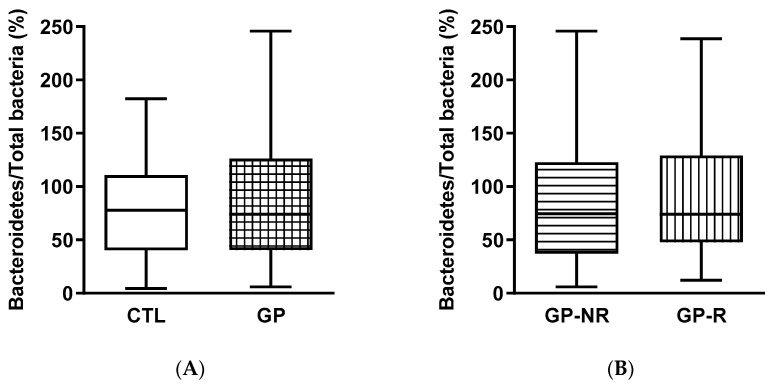

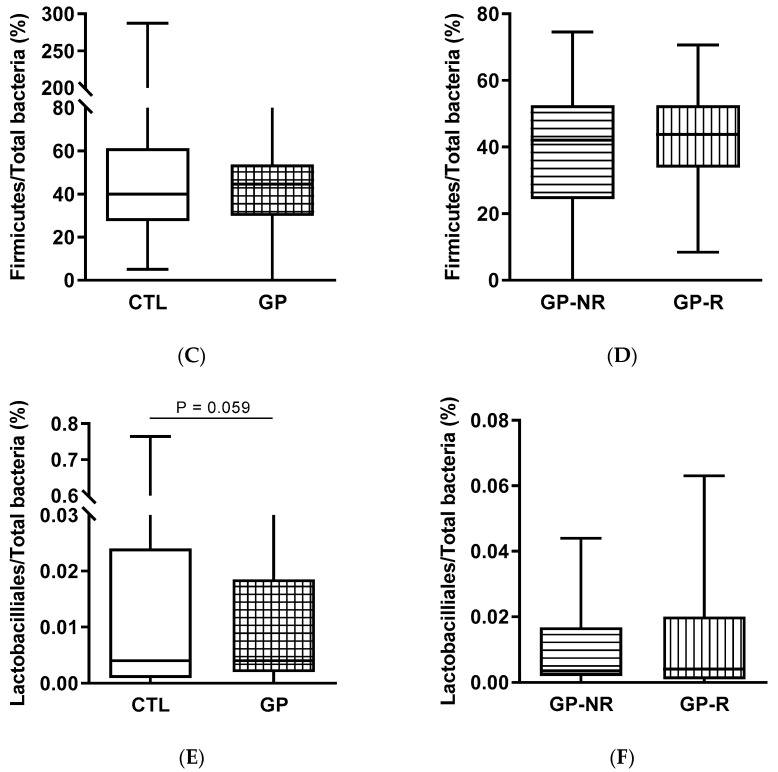
Excreted gut bacteria expressed as percentages of total bacteria in feces from subjects at high cardiometabolic risk participating in the clinical trial on grape pomace (GP) supplementation: CTL, control period without supplementation; GP, after GP supplementation; GP-NR, non-responders to GP supplementation; GP-R, responders to GP supplementation. (**A**,**B**): Bacteroidetes; (**C**,**D**): Firmicutes; (**E**,**F**): Lactobacilliales.

**Figure 2:**
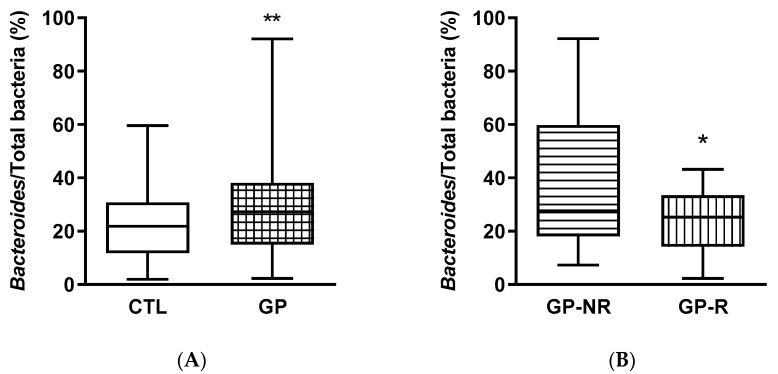

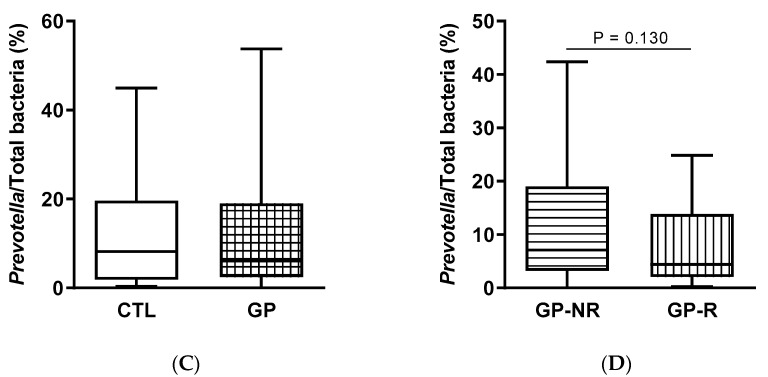
Excreted gut bacteria expressed as percentages of total bacteria in feces from subjects at high cardiometabolic risk participating in the clinical trial on grape pomace (GP) supplementation: CTL, control period without supplementation; GP, after GP supplementation; GP-NR, non-responders to GP supplementation; GP-R, responders to GP supplementation. (**A**,**B**): *Bacteroides*; (**C**,**D**): *Prevotella*. *, *p*-value < 0.05; **, *p*-value < 0.01.

The proportions of selected bacterial phyla, order, and genus of gut microbiota were evaluated at the beginning and the end of the supplementation (Figure 1 and Figure 2). There were no differences between the groups in the Bacteroidetes and Firmicutes phyla. The GP supplementation tended to reduce (*p* = 0.059) the proportion of the order Lactobacilliales (Figure 1E) with respect to the CTL period, while these proportions were similar in GP-R and GP-NR groups.

The population of the genus *Bacteroides* was significantly (*p* < 0.01) increased by the GP intervention (Figure 2A). This difference is attributable exclusively to the GP-NR group, as the proportion of *Bacteroides* in the GP-R group was significantly (*p* < 0.05) lower than that in the GP-NR group (Figure 2B) and similar to that in the CTL period. The proportions of the genus *Prevotella* were similar between the CTL and GP periods, while there was a tendency (*p* = 0.130) to a reduction in the proportion of *Prevotella* in the R group (Figure 2D).

Fecal concentration of SCFAs were similar between periods, with the exception of isovaleric acid, that was significantly (*p* < 0.05) lower after GP supplementation than in CTL period (Table 3).

## 4. Discussion

Polyphenols are dietary bioactive compounds able to induce subtle modifications in many physiological processes [41]. When associated with insoluble dietary fibers as it occurs in GP, their effects may be additive to those of the dietary fiber [42]. This diversity of mechanisms of action makes it difficult to elucidate the processes eventually involved in the health effects associated with polyphenol-rich dietary fibers such as GP. Gut microbiota may play a prominent role in the action of GP. As clinical trials in the field of polyphenols and microbiota are still scarce, we explored the effect of GP supplementation (a polyphenol-rich product containing both extractable and non-extractable polyphenols) on the populations of some significant subgroups of gut microbiota in subjects at cardiometabolic risk.

As observed in a previous study, where a GP extract was administered to healthy women [28], GP did not cause significant modifications in the major microbiota subgroups tested in subjects supplemented for 6 weeks. This suggests that the observed reduction of insulin levels in overweight subjects at cardiometabolic risk by GP was not induced by changes in the major subgroups of gut microbiota. A tendency (*p* = 0.059) towards a decrease in the proportion of Lactobacilliales was observed after the supplementation. Although this effect may seem surprising according to the expected beneficial effects of GP and the traditional consideration of this order as a whole as beneficial bacteria, several studies have shown that this relationship is not so unequivocal. Thus, an increase in the Lactobacillaceae family was observed in mice fed industrially-generated trans-fatty acids, an effect further confirmed by in vitro fermentation of these compounds by the family Lactobacillaceae and the genus *Lactobacillus* [43]. A tendency towards an increase in Lactobacilliales was observed in subjects with Behcet’s syndrome, a systemic inflammatory condition [44] and, recently, an increase in the genus *Lactobacillus* has been reported in diabetic rats, being significantly associated with an increase in body weight and adverse modification in biomarkers of glucose homeostasis and inflammation [45]. While it is still not clear whether the increase in Lactobacilliales in these adverse situations is a cause or a consequence, after observing an increase in several *Lactobacillus* spp. in an animal model of steatohepatitis, the authors suggested that this was due to their involvement in bile acid metabolism [46]. Further studies on the health effects of specific bacterial species belonging to Lactobacilliales are thus needed.

GP supplementation did not show any significant effect on the major phyla (Bacteroidetes and Firmicutes) of gut microbiota. Then, we examined the populations of the main genera within Bacteroidetes, *Prevotella* and *Bacteroides,* because the ratio between these two subgroups has been related to the intake of dietary fiber [47]. The populations of *Prevotella* were also similar in the controls and supplemented groups while *Bacteroides* significantly increased after GP supplementation. Similarly, the populations of *Bacteroides* were increased by other grape-derived products such as grape seed proanthocyanidins [48] and wine [49]. *Bacteroides* are particularly well-adapted to harsh environments through several biochemical tools, such as enzymes capable of metabolizing many diet- and host-derived polysaccharides, cytochrome bd oxidase to tolerate oxygen, and glycosyl transferases that generate a vast number of cell surface structures [50]. Our results are suggesting that *Bacteroides* are particularly equipped to degrade and use GP to foster their growth while *Prevotella* spp. remain mostly irresponsive. One of the main mediators that link gut microbiota and host metabolism are the SCFA, by fermenting indigestible dietary constituents. The changes in gut microbiota are consistent with the outcome of SCFA analysis (Table 3), which did not show any significant difference between groups.

A closer observation of the results from the human experiment revealed that the increase in the relative population of *Bacteroides* was only evident in the GP-NR group (Figure 2B). The subjects in the GP-NR group were those with significantly lower fasting plasma insulin concentration compared to those in the GP-R group, which upon GP supplementation had their insulin levels reduced (Ramos-Romero et al., *submitted*). These results evidence that the possible relationship between the *Bacteroides* sensitivity to GP and insulin responsiveness deserves further attention, including a more thorough examination at the species level.

The main limitations of this study arise from the lack of an appropriate placebo in the study design, as well as microbiota evaluation at species level. Future studies using next-generation sequencing techniques could complete the presented results. In contrast, the main strengths of the study are a cross-over design focused on overweight or obese subjects at cardiometabolic risk, as well as the comparative evaluation of microbiota between responders and non-responders s to GP supplementation.

## 5. Conclusions

Results from a randomized cross-over clinical trial suggest that a reduction of insulin levels in overweight subjects at cardiometabolic risk by GP is not induced by changes in the major subgroups of gut microbiota. Those subjects non-responsive to GP supplementation experienced a significant increase in the relative population of the genus *Bacteroides*. Further studies at the species level should shed more light on the biological significance of the changes induced by GP in the gut microbiota.

## Figures and Tables

**Table 1:** Quantitative real-time PCR (qPCR) primers and conditions.

Target Bacteria	Annealing Temperature (°C)	Sequences (5′-3′)	Positive Control	Reference
Total Bacteria	65	F: ACT CCT ACG GGA GGC AGC AGT	(^a^)	[35]
		R: ATT ACC GCG GCT GCT GGC		
Bacteroidetes	62	F: ACG CTA GCT ACA GGC TTA A	*Bacteroides fragilis*	[36]
		R: ACG CTA CTT GGC TGG TTC A		
Firmicutes	52	F: CTG ATG GAG CAA CGC CGC GT	*Ruminococcus productus*	[37]
		R: ACA CYT AGY ACT CAT CGT TT		
Lactobacillales	60	F: AGC AGT AGG GAA TCT TCC A	*Lactobacillus acidophylus*	[38]
		R: CAC CGC TAC ACA TGG AG		
*Bacteroides*	60	F: GGT TCT GAG AGG AGG TCC C	*Bacteroides fragilis*	[39]
		R: GCT GCC TCC CGT AGG AGT		
*Prevotella*	60	F: CAG CAG CCG CGG TAA TA	*Prevotella copri*	[39]
		R: GGC ATC CAT CGT TTA CCG T		

^a^ Positive control of total bacteria was the same as those the result was rated with.

**Table 2:** Evolution in cardiometabolic parameters in subjects at high cardiometabolic risk participating in the clinical trial on grape pomace (GP) supplementation, classified according to their modifications in insulin response.

Parameter	Pre-GP Supplementation		Post-GP Supplementation (Variation, %)				*p*-Value *
	Whole Sample (*n* = 49)		Non-Responders (*n* = 26)		Responders (*n* = 23)		
	Mean	S.E.M.	Mean	S.E.M.	Mean	S.E.M.	
Body mass index (kg/m^2^)	31	1	−0.2	0.3	−0.1	0.2	0.618
Abdominal perimeter (cm)	103	2	0.2	0.4	−0.5	0.6	0.591
Total body fat (%)	31	1	0.1	0.8	−1.7	0.9	0.249
Abdominal fat (%)	103	2	−0.3	1.1	0.1	0.8	0.880
Systolic blood pressure (mm Hg)	120	17	−2	3	2	2	0.322
Diastolic blood pressure (mm Hg)	84	12	−1	3	2	2	0.809
Glucose (mg/dL)	98	2	7	2	1	2	0.540
Insulin (µU/mL)	8.9	1.9	82	10	−40	4	**<0.00001**
HOMA-IR	2.1	0.4	100	10	−40	4	**<0.00001**
Triglycerides (mg/dL)	147	21	22	8	−2	5	0.595
Total cholesterol (mg/dL)	341	48	−3	2	−5	2	0.353
HDL cholesterol (mg/dL)	47	7	0.0	2	4	2	0.200
LDL cholesterol (mg/dL)	121	17	−6	2	−8	2	0.873
Fibrinogen (mg/dL)	340	50	−0.4	2	−4	2	0.567
Plasma uric acid (mg/dL)	6	1	3	2	−0.3	1	0.548
Urine uric acid (mg/g creatinine)	490	70	−5	7	5	6	0.318

* signficant differences (<0.05) marked in bold; S.E.M., standard error of the mean.

**Table 3:** Short-chain fatty acid (SCFA) concentration in feces from subjects at high cardiometabolic risk participating in the clinical trial on GP supplementation: CTL, control period without supplementation; GP, after GP supplementation; GP-NR, non-responders to GP supplementation; GP-R, responders to GP supplementation.

Compound	CTL		GP		*p*-Value	GP-NR		GP-R		*p*-Value
	Mean	SEM	Mean	SEM		Mean	SEM	Mean	SEM	
Acetic acid	120	20	120	10	0.602	110	20	118	20	0.879
Propionic acid	55	8	56	7	0.972	60	10	49	9	0.438
Isobutyric acid	6.9	0.5	6.9	0.7	0.940	6.8	0.6	7.1	1.4	0.852
Butyric acid	38	5	31.6	4.1	0.352	31	6	32	6	0.943
Isovaleric acid	7.1	0.5	5.7	0.4	0.023 *	6.0	0.6	5.2	0.6	0.344
Valeric acid	6.8	0.8	5.8	0.7	0.435	6.1	1.0	5.6	1.1	0.731

* signficant differences (<0.05) marked in bold.

## Supplementary Materials

The following are available online at https://www.mdpi.com/2304-8158/9/9/1279/s1, Table S1: Basal individual values for Metabolic Syndrome factor in subjects at high cardiometabolic risk participating in the clinical trial on grape pomace supplementation.

## Abbreviations

HOMA-IR	homeostatic model assessment for insulin resistance
MetS	metabolic syndrome
OGT	oral glucose tolerance test
GP	grape pomace
