# Supplementary material for: Modifications of Gut Microbiota after Grape Pomace Supplementation in Subjects at Cardiometabolic Risk: A Randomized Cross-Over Controlled Clinical Trial
Source: Foods. 2020 Sep 11;9(9):1279. doi: 10.3390/foods9091279 (PMC7555163; doi:10.3390/foods9091279)
Supplement: Supplementary file 1 [file foods-09-01279-s001.pdf]

Electronic supplementary material:

Table S1. Basal individual values for Metabolic Syndrome factor in subjects at high cardiometabolic risk participating in the clinical trial on grape pomace supplementation.

Subject [1]	Sex	Age (year)	BMI (kg/m^2)	SBP (mmHg)	DBP (mmHg)	Glucose (mg/dL)	HDL- chol (mg/dL)	Triglycerides (mg/dL)
1	F	57	25.09	124.75	80.50	92.5	43.5	253.5
2	M	50	32.03	110.00	86.00	79.5	39.0	161.5
3	M	54	25.47	121.50	82.00	102.5	51.0	168.0
4	F	48	29.49	134.00	85.25	84.5	43.5	145.0
5	M	57	30.19	124.25	74.25	115.0	37.0	232.0
6	M	41	25.56	99.00	62.25	93.5	45.0	135.5
7	F	54	33.61	135.00	94.00	103.0	59.5	169.5
8	M	25	27.89	134.00	80.50	93.0	38.5	318.5
10	M	37	28.48	113.84	80.40	101.0	60.0	79.5
11	M	53	37.94	146.50	91.50	105.5	67.5	109.0
12	M	42	27.90	117.25	79.00	98.5	42.0	210.5
13	M	44	37.40	118.50	85.00	107.5	43.5	206.0
14	F	19	27.02	113.50	82.75	92.5	64.5	143.5
15	M	59	28.18	124.34	93.84	113.0	35.5	237.5
16	F	46	28.96	95.50	69.75	89.0	49.5	73.0
17	F	56	28.78	120.75	82.50	98.0	58.0	195.0
18	F	30	30.48	100.00	67.75	97.0	39.5	96.5
19	M	21	31.94	116.00	86.75	92.0	36.5	106.0
20	M	39	25.23	134.00	101.13	92.5	37.0	119.0
21	M	49	38.89	118.75	84.75	104.5	35.5	306.0
22	M	22	28.34	121.75	74.25	95.5	53.5	59.0
23	F	62	32.73	130.50	85.50	135.0	48.0	NA
24	M	22	29.85	120.00	78.25	96.0	47.5	79.5
25	M	42	42.70	122.75	83.75	87.0	43.0	106.5
26	F	44	26.46	140.00	97.25	99.5	48.5	88.5
27	M	35	28.55	125.50	88.25	98.0	54.5	77.0
28	M	42	30.15	111.00	79.25	102.0	48.5	194.5
29	F	51	25.95	122.75	88.50	110.5	71.0	119.5
30	F	31	32.97	93.25	69.00	88.5	51.0	76.5
31	F	38	36.63	103.75	73.00	101.5	44.5	144.5
32	M	42	32.32	105.50	76.25	101.5	42.5	131.0
33	M	52	29.75	118.75	85.00	109.0	41.5	168.5
34	F	24	36.45	99.25	70.00	90.5	42.0	81.5
35	F	28	25.49	101.50	74.00	82.5	51.5	235.5
36	F	31	26.51	96.50	69.50	83.5	39.5	100.0
37	F	37	50.55	109.50	82.75	103.0	59.0	94.0
38	M	33	30.45	110.00	78.75	84.5	29.5	244.0
39	M	49	29.74	123.50	80.00	99.0	50.5	100.5
40	F	33	35.51	108.00	80.75	116.5	65.0	124.0
41	F	65	27.30	119.75	73.00	103.0	81.0	64.0
43	F	50	45.06	138.38	105.25	96.0	65.0	168.0
44	M	44	37.83	117.50	84.25	113.0	35.0	163.0

45	F	43	24.93	109.00	77.50	87.0	44.5	108.5
46	M	53	31.86	151.25	98.25	102.0	60.5	38.5
48	F	49	25.56	122.75	86.50	102.5	61.5	62.0
49	M	54	25.37	115.00	81.50	97.5	32.0	269.5
50	M	38	25.96	119.50	91.00	108.0	32.0	123.0
51	F	44	35.47	114.00	92.00	101.0	53.0	140.0
52	M	48	25.62	120.50	81.00	84.0	52.0	118.0

[1] Original code assigned to the subject (52 participants originally enrolled, data shown here correspond to the 49 subjects completing the study). F, female; M, male; SBP, systolic blood pressure; DBP, diastolic blood pressure; chol, cholesterol.
